# Directed Interband Response at Null Biorthogonal Quantum-Geometric Components

**DOI:** 10.3390/e28080936

**Published:** 2026-08-21

**Authors:** Xinyi Xie, Jia-Ning Zhu, Bo Wan

**Affiliations:** School of Science, Jiangsu University of Science and Technology, Zhenjiang 212100, China

**Keywords:** non-Hermitian systems, quantum geometric tensor, biorthogonal geometry, two-band models, response vertices, exceptional points

## Abstract

Biorthogonal quantum geometry is often read through scalar tensor components. In non-Hermitian bands, however, the biorthogonal contraction can lose the ordering of the left-right interband matrix elements from which a scalar component is formed. We study this information loss for spectrally separated, diagonalizable two-band Bloch Hamiltonians. For a specified control parameter, the Hamiltonian variation defines a local response vertex. In the instantaneous biorthogonal eigenbasis, the interband part of this vertex is completely specified by two ordered matrix elements, whereas the corresponding equal-parameter scalar QGT component retains only their product. This separation leads to a local classification of interband vertices into no-interband, Hermitian-locked, generic complex-transverse, and complex-null cases. On a complex-null branch, the scalar component can vanish even though one ordered interband matrix element remains nonzero. We identify this as a local chiral-vertex mechanism in a vertex-resolved geometric response kernel, distinct from generic non-Hermiticity or exceptional-point proximity. Nonreciprocal SSH, a two-dimensional complex-spin–orbit lattice, and a $k_{z}$-only chiral ladder stack realize the same mechanism in one, two, and three dimensions, while diagonal and gain–loss-like vertices provide nonselective comparisons.

## 1. Introduction

Non-Hermitian Hamiltonians provide effective descriptions of open, lossy, active, and classical-wave systems. They have become a standard language for exceptional points, non-orthogonal eigenvectors, non-Bloch spectra, and skin-effect physics [1,2,3,4,5,6,7,8,9,10,11]. In these systems, complex spectra, exceptional degeneracies, non-Bloch band structure, and skin accumulation are central spectral and boundary phenomena [8,9,11,12,13,14,15]. At the same time, non-Hermitian eigenstates form biorthogonal geometric frames whose variation with momentum and external parameters is itself a geometric structure of interest [16,17,18,19,20,21]. The quantum geometric tensor provides a local description of this eigenstate geometry and its associated response content.

In Hermitian bands, the quantum geometric tensor unifies the Fubini–Study metric and Berry curvature, and it appears in Berry-phase, polarization, Wannier-localization, optical, and transport-response formulations [22,23,24,25,26,27,28,29,30]. Direct measurements in photonic and polaritonic settings provide an experimental reference point for this geometric tensor [31,32]. Recent plasmonic lattices show how quantum metric and non-Hermitian Berry-curvature signatures can be accessed in structured optical platforms [20,21]. In non-Hermitian systems, complex geometric phases and holonomies arise naturally in dissipative settings [33], and two-level exceptional-point geometry has been described using complex Bloch-sphere and complex-monopole language [34]. Recent work has connected biorthogonal geometry to complex or pseudo-Riemannian metrics, lightlike paths, exceptional-point wave-packet dynamics, experimentally accessible metric signatures, adiabatic-generator fluctuations, and nonlinear response [16,17,18,19,35,36,37,38,39]. The same language is naturally adjacent to biorthogonal quantum mechanics, pseudo-Hermitian state-space geometry, and information geometry [40,41,42,43]. These developments motivate using the quantum geometric tensor as a local geometric response object. A tensor component, however, is an inner-product contraction. In Hermitian bands this contraction is tied to the usual Hilbert-space metric, and the two off-diagonal matrix elements are conjugate partners. In a non-Hermitian band the contraction is instead biorthogonal: left and right eigenvectors define dual frames that are generally independent of Hermitian conjugation. This raises the basic question studied below: what information is retained, and what information is discarded, when non-Hermitian eigenstate geometry is reduced to a scalar biorthogonal QGT component?

In this work, we answer this question by keeping the physical control direction explicit. A scalar component is tied to a specified variation of the local Hamiltonian, such as momentum, flux, hopping, mass, strain, or gain and loss. We write the corresponding response vertex as $F_{v}$. Instead of interpreting the scalar component alone, we compare it with the left-right matrix elements of $F_{v}$ in the instantaneous biorthogonal eigenbasis. For a spectrally separated, diagonalizable two-band block, this gives a local classification of the two off-diagonal vertex coordinates. In the Hermitian limit, the opposite off-diagonal matrix elements are conjugate-locked. The non-Hermitian case admits complex-null interband vertices for which the scalar component can vanish, although one ordered matrix element remains finite. The product identity itself follows from two-band biorthogonal algebra. The new step here is to classify the physical vertex that enters this product: different points in $g_{\pm }$-space can give the same scalar value while retaining different ordered matrix-element content.

The two-band case makes this statement explicit. After the band-diagonal part of the local Hamiltonian variation is separated, the remaining interband information consists of two ordered matrix elements, one for each left–right ordering. The scalar QGT component along the specified drive retains only their product. Thus, the local problem is to classify where the physical perturbation lies in the two-complex-dimensional interband vertex space. Generic points give a finite scalar component. On the complex-null branches, however, one ordered matrix element is suppressed, so the product vanishes while the reverse ordering can remain finite. Here, by “null” we mean that the scalar geometric component for a specified physical perturbation vanishes in the local nondegenerate two-band block. The underlying response content is then read from the ordered interband matrix elements before the scalar contraction is taken.

This paper is organized as follows. Section 2 defines the biorthogonal response vertex associated with a physical control parameter, derives the two-band product identity, and rewrites the scalar component in terms of the two interband coordinates $g_{+}^{\left(v\right)}$ and $g_{-}^{\left(v\right)}$. This keeps the two ordered factors separate before the scalar contraction is taken. Section 3 classifies this local vertex space into no-interband, Hermitian-locked, generic complex-transverse, and complex-null cases. Section 4 studies physical realizations of the response-relevant classes in Bloch models. Nonreciprocal SSH, a two-dimensional complex-spin–orbit lattice, and a $k_{z}$-only ladder stack give one-, two-, and three-dimensional realizations of the complex-null branch. Diagonal and gain–loss-like vertices do not isolate a single ordered matrix element even in non-Hermitian bands. Section 5 discusses the scope of the local response mechanism. It separates the Bloch vertex classification used here from topological, skin-effect, and non-Bloch descriptions of non-Hermitian bands [12,13,14,44,45,46,47,48,49,50,51,52,53,54,55,56,57,58,59,60].

## 2. Biorthogonal Response Vertices and Two-Band Product Identity

We use the standard biorthogonal formulation of non-Hermitian band problems, where right eigenvectors and left eigenbras form dual bases rather than Hermitian–conjugate pairs [1,40,41,42]. This construction does not require a protecting symmetry of *H*; it requires diagonalizability of the band block in the parameter region considered. Symmetry-constrained subclasses, such as PT-symmetric or pseudo-Hermitian Hamiltonians, impose additional structure but are not assumptions of the derivation [42,61]. In a diagonalizable, nondegenerate region of parameter space, we define the right and left eigenvectors by

(1) $$H|u_{n}^{R}\rangle =E_{n}|u_{n}^{R}\rangle ,\;\langle u_{n}^{L}|H=E_{n}\langle u_{n}^{L}|,$$

and impose the biorthonormality and completeness conditions

(2) $$\langle u_{m}^{L}|u_{n}^{R}\rangle =\delta_{mn},\;I=\sum_{n}|u_{n}^{R}\rangle \langle u_{n}^{L}|.$$

The physical vertex associated with a parameter *v* is

(3) $$F_{v}=\partial_{v}H.$$

Differentiating Equation (1) and projecting with $\langle u_{m}^{L}|$, m≠n, gives

(4) $$A_{mn}^{\left(v\right)}=\langle u_{m}^{L}|\partial_{v}u_{n}^{R}\rangle =\frac{\langle u_{m}^{L}|F_{v}|u_{n}^{R}\rangle }{E_{n}-E_{m}}.$$

The order of the labels is part of the local response information: $A_{mn}^{\left(v\right)}$ connects the right eigenstate *n* to the left eigenbra *m* through $F_{v}$. In a non-Hermitian problem the reverse amplitude $A_{nm}^{\left(v\right)}$ is not fixed by complex conjugation of $A_{mn}^{\left(v\right)}$.

Under a biorthogonal rescaling

(5) $$|u_{n}^{R}\rangle \;\to e^{\chi_{n}}|u_{n}^{R}\rangle ,\;\langle u_{n}^{L}|\;\to e^{-\chi_{n}}\langle u_{n}^{L}|,$$

the off-diagonal element transforms as

(6) $$A_{mn}^{\left(v\right)}\to e^{\chi_{n}-\chi_{m}}A_{mn}^{\left(v\right)}.$$

Consequently, because the rescaling factors are nonzero, the zero or nonzero status of each off-diagonal element is invariant, and products of opposite off-diagonal elements are invariant. Absolute magnitudes away from zeros depend on the normalization of the biorthogonal eigenvectors. Ratios of finite magnitudes are therefore fixed-convention quantities rather than gauge-invariant numbers. In the numerical work below, the right eigenvectors are the columns of a matrix *R*, and the left bras are the rows of $R^{-1}$, enforcing Equation (2). Exact exceptional points, where the biorthogonal basis is defective, are excluded.

Consider a spectrally separated two-band block with eigenvalues $E_{\pm }=d_{0}\pm \varepsilon$, ε≠0. For the upper band, define

(7) $$P_{+}=|u_{+}^{R}\rangle \langle u_{+}^{L}|,\;1-P_{+}=|u_{-}^{R}\rangle \langle u_{-}^{L}|.$$

The second equality uses the two-band completeness relation in Equation (2). The scalar biorthogonal component along *v* is

(8) $$Q_{vv}^{\left(+\right)}=\langle \partial_{v}u_{+}^{L}|\left(1-P_{+}\right)|\partial_{v}u_{+}^{R}\rangle .$$

Here, $\langle \partial_{v}u_{+}^{L}|$ means $\partial_{v}\langle u_{+}^{L}|$; the derivative acts on the left bra itself. Inserting the complementary projector gives

(9) $$Q_{vv}^{\left(+\right)}=\langle \partial_{v}u_{+}^{L}|u_{-}^{R}\rangle \langle u_{-}^{L}|\partial_{v}u_{+}^{R}\rangle .$$

Differentiating $\langle u_{+}^{L}|u_{-}^{R}\rangle =0$ yields

(10) $$\langle \partial_{v}u_{+}^{L}|u_{-}^{R}\rangle =-\langle u_{+}^{L}|\partial_{v}u_{-}^{R}\rangle ,$$

and therefore

(11) $$Q_{vv}^{\left(+\right)}=-A_{+-}^{\left(v\right)}A_{-+}^{\left(v\right)}.$$

Equation (11) is the two-band product identity. The scalar component retains the invariant product of the two ordered matrix elements but not which ordered matrix element is small or zero.

To make the local classification explicit, expand the same physical vertex in the instantaneous eigenbasis. We use $\tau_{z}=|u_{+}^{R}\rangle \langle u_{+}^{L}|-|u_{-}^{R}\rangle \langle u_{-}^{L}|$, $\tau_{+}=|u_{+}^{R}\rangle \langle u_{-}^{L}|$, and $\tau_{-}=|u_{-}^{R}\rangle \langle u_{+}^{L}|$. Write

(12) $$F_{v}=a_{v}I+b_{v}\tau_{z}+g_{+}^{\left(v\right)}\tau_{+}+g_{-}^{\left(v\right)}\tau_{-},$$

where

(13) $$g_{+}^{\left(v\right)}=\langle u_{+}^{L}|F_{v}|u_{-}^{R}\rangle ,\;g_{-}^{\left(v\right)}=\langle u_{-}^{L}|F_{v}|u_{+}^{R}\rangle .$$

Using Equation (4),

(14) $$A_{+-}^{\left(v\right)}=-\frac{g_{+}^{\left(v\right)}}{2\varepsilon },\;A_{-+}^{\left(v\right)}=\frac{g_{-}^{\left(v\right)}}{2\varepsilon },$$

where the generally complex gap 2ε fixes the common energy denominator for this two-band block. The amplitude scale is therefore gap-dependent, whereas the chiral zero structure is determined by $g_{\pm }^{\left(v\right)}$. The product identity then gives

(15) $$Q_{vv}^{\left(+\right)}=\frac{g_{+}^{\left(v\right)}g_{-}^{\left(v\right)}}{4\varepsilon^{2}}.$$

The identity and longitudinal pieces $a_{v}I+b_{v}\tau_{z}$ have no interband geometry. For a fixed physical vertex, the local two-band interband content entering the scalar component $Q_{vv}^{\left(+\right)}$ is specified by the ordered pair $\left(g_{+}^{\left(v\right)},g_{-}^{\left(v\right)}\right)$.

For two-band Hamiltonians $H=d_{0}I+d\cdot \sigma$, the same QGT has a compact *d*-vector form. Choose a local branch of $\varepsilon^{2}=d\cdot d$, where the dot product is complex bilinear, and set n=d/ε. The projectors are

$$P_{s}=\frac{1}{2}\left(I+s\;n\cdot \sigma \right),\;s=\pm .$$

Using $Q_{\mu \nu }^{\left(s\right)}=\mathrm{Tr}\;\left[P_{s}\left(\partial_{\mu }P_{s}\right)\left(1-P_{s}\right)\left(\partial_{\nu }P_{s}\right)\right]$, together with n·n=1, $n\cdot \partial_{\mu }n=0$, and the Pauli trace identities, gives

(16) $$Q_{\mu \nu }^{\left(s\right)}=\frac{1}{4}\left[\partial_{\mu }n\cdot \partial_{\nu }n+is\;n\cdot \left(\partial_{\mu }n\times \partial_{\nu }n\right)\right].$$

Equivalently,

(17) $$\begin{array}{cc} Q_{\mu \nu }^{\left(s\right)}= & \frac{1}{4}\left[\frac{\partial_{\mu }d\cdot \partial_{\nu }d}{\varepsilon^{2}}-\frac{\left(d\cdot \partial_{\mu }d\right)\left(d\cdot \partial_{\nu }d\right)}{\varepsilon^{4}}\right.\\ \; & \left.+\frac{is\;d\cdot (\partial_{\mu }d\times \partial_{\nu }d)}{\varepsilon^{3}}\right]. \end{array}$$

For μ=ν=v, the last term vanishes and the upper-band component becomes

(18) $$Q_{vv}^{\left(+\right)}=\frac{1}{4}\left[\frac{\partial_{v}d\cdot \partial_{v}d}{\varepsilon^{2}}-\frac{{\left(d\cdot \partial_{v}d\right)}^{2}}{\varepsilon^{4}}\right].$$

These expressions are valid away from band degeneracies and defective points. They connect the vertex classification to the usual two-band *d*-vector QGT formula used in the model calculations, but they have already combined the two ordered interband matrix elements. We therefore return below to the vertex coordinates $\left(g_{+}^{\left(v\right)},g_{-}^{\left(v\right)}\right)$, where the two factors entering Equation (15) remain separate.

## 3. Local Classification and Directed Response

**Figure 1:**
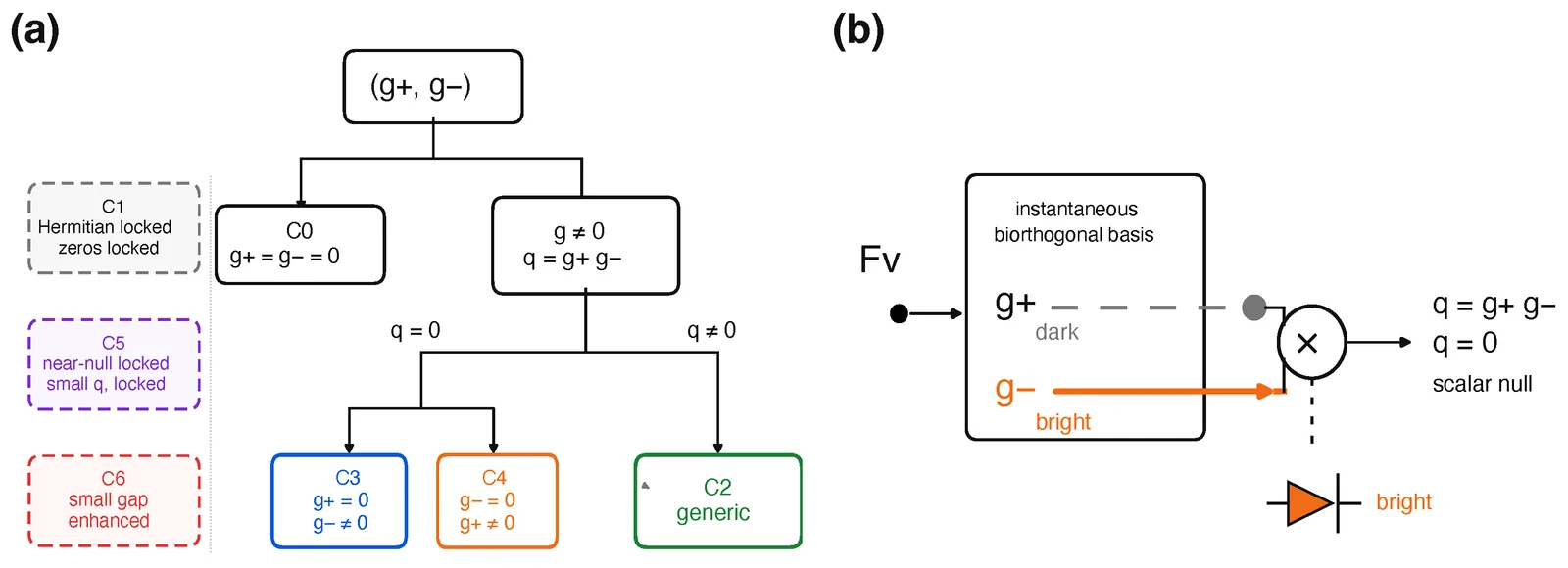
$F_{v}/g_{\pm }$-space classification of local two-band vertices. (**a**) The ordered interband coordinates $\left(g_{+},g_{-}\right)$ separate the no-interband, Hermitian-locked, generic complex-transverse, and chiral branches. (**b**) The scalar geometric component keeps only the product $q=g_{+}g_{-}$, so the two chiral branches fold to the same scalar-null locus q=0. The labels C5 and C6 denote common near-null situations rather than additional algebraic strata. The dark and bright labels in panel (**b**) denote zero and nonzero ordered matrix elements in the displayed local basis; finite color contrast is convention-fixed. The figure describes the local vertex geometry; band topology and boundary spectra enter through additional structure.

Equations (12)–(15) give a local classification of the response vertex. After removing the identity and longitudinal directions, a local two-band vertex is represented in the instantaneous biorthogonal basis by the ordered pair $\left(g_{+},g_{-}\right)\in C^{2}$. The scalar component is the product map $q=g_{+}g_{-}$. The zero/nonzero branch structure of this pair gives the invariant part of the classification, while finite contrasts away from zeros are quoted only in the fixed biorthogonal convention stated above. Figure 1 shows this local vertex-space geometry.

The algebraic classes and their physical readings are summarized in Table 1. Figure 1a displays the logic of the local classification: the no-interband, Hermitian-locked, generic, and two chiral cases occupy distinct parts of the interband vertex space. Figure 1b shows the scalar contraction: the product map keeps $q=g_{+}g_{-}$, so the two chiral branches are indistinguishable at the level of a scalar-null readout. Cases 0–4 are the local vertex-space strata after the band-diagonal components have been removed. The near-null situations called C5 and C6 in Figure 1 are discussed after the table because they are not additional algebraic strata.

The first distinction is between vertices with finite interband content and vertices with zero interband content. Class 0 includes a pure identity vertex and the longitudinal part of a two-band Hamiltonian. Such a perturbation changes a common energy offset or the separation of the two eigenvalues while leaving the biorthogonal eigenvectors fixed. In this class, a null scalar component has a conventional interpretation: along that parameter, the interband geometric response vanishes.

The next class is the Hermitian-locked slice. If both *H* and $F_{v}$ are Hermitian, one may choose the usual unitary eigenbasis, and the two off-diagonal vertex matrix elements are conjugate-locked. Under a general biorthogonal rescaling, their finite relative magnitudes depend on the chosen normalization, but the zero statement remains invariant: $g_{+}=0$ if and only if $g_{-}=0$. Thus one off-diagonal element cannot vanish while the reverse element remains finite. This is the familiar Hermitian situation: the scalar component is small only when the underlying interband matrix element is small.

Class 2 is the generic complex-transverse region. Both ordered matrix elements are nonzero, and the scalar component is finite in general. Because left and right eigenvectors are independent biorthogonal objects, the two ordered matrix elements can have different magnitudes in a chosen normalization. This region can therefore show matrix-element asymmetry without making the scalar product vanish.

Classes 3 and 4 are the complex-null, or chiral, branches. On one branch, $g_{+}=0$ while $g_{-}\neq 0$, and on the other branch the labels are interchanged. From Equation (15),

$$Q_{vv}^{\left(+\right)}=0,\;A_{+-}^{\left(v\right)}=0,\;A_{-+}^{\left(v\right)}\neq 0$$

on the first branch, with the two ordered labels interchanged on the second branch. In this case the scalar component is null because the product contains a zero factor while the local interband content remains finite. The scalar component alone does not specify which ordered matrix element has vanished.

Here, by “null” we mean that a particular scalar geometric component of a local two-band model is zero or small. The $g_{\pm }$-space classification decides whether this null component comes from no interband geometry, Hermitian locking, or a chiral product with one finite ordered matrix element. This usage is tied to the specified scalar QGT component and should be distinguished from lightlike directions in a pseudo-Riemannian state-space metric.

Two common near-null situations should be kept separate from the algebraic classification. First, a scalar component can be small without selecting one ordered matrix element, as in the diagonal gain/loss-type stacking vertex used below. Second, a small gap or an EP-proximate region can enhance both ordered matrix elements through the energy denominator. The mechanism studied here is instead proximity to a chiral branch in the local interband vertex space.

For the present local problem, model dependence enters through the physical path $F_{v}\left(\lambda \right)$ in this vertex space. The Bloch Hamiltonians below show how lattice perturbations generate such paths. Chiral hopping or stacking vertices approach the complex-null branch, whereas diagonal stacking or reciprocal gain-loss vertices can make the scalar component small without selecting a single ordered matrix element.

## 4. Model Realization

The local classification is tied to a physical Hamiltonian once the perturbation is specified. The Bloch Hamiltonians below are representative realizations of the vertex classes, not an exhaustive catalog of models. In each case, we track the physical perturbation $F_{v}$ and its interband projection in the local biorthogonal eigenbasis. The numerical results compare the scalar component with the two ordered interband matrix elements along the same Bloch-space path. All model calculations are made in parameter regions where the two-band Bloch Hamiltonian is diagonalizable. They describe local vertices of the infinite periodic system; open-boundary spectra and non-Bloch wave functions require additional boundary and readout information [15]. The same local identity is used in every dimension: a scalar geometric component can be small because one ordered interband factor is suppressed, not because all interband matrix elements are small.

### 4.1. One-Dimensional Nonreciprocal SSH/Rice-Mele Chain

The one-dimensional lattice example is

(19) $$H_{\mathrm{nr}}\left(k\right)=\left(\begin{array}{cc} m & t_{R}+t_{2}e^{-ik}\\ t_{L}+t_{2}e^{ik} & -m \end{array}\right).$$

This is the Bloch form underlying nonreciprocal SSH and related topolectrical, mechanical, and non-Bloch platforms [46,49,50,51,62,63,64,65]. The calculation below uses the periodic Bloch Hamiltonian to evaluate the local vertex. Open-boundary skin modes and non-Bloch spectra enter only when a finite-boundary response problem is specified. The relevant local object is the hopping modulation

$$F_{t_{R}}=\partial_{t_{R}}H_{\mathrm{nr}}=\left(\begin{array}{cc} 0 & 1\\ 0 & 0 \end{array}\right).$$

**Figure 2:**
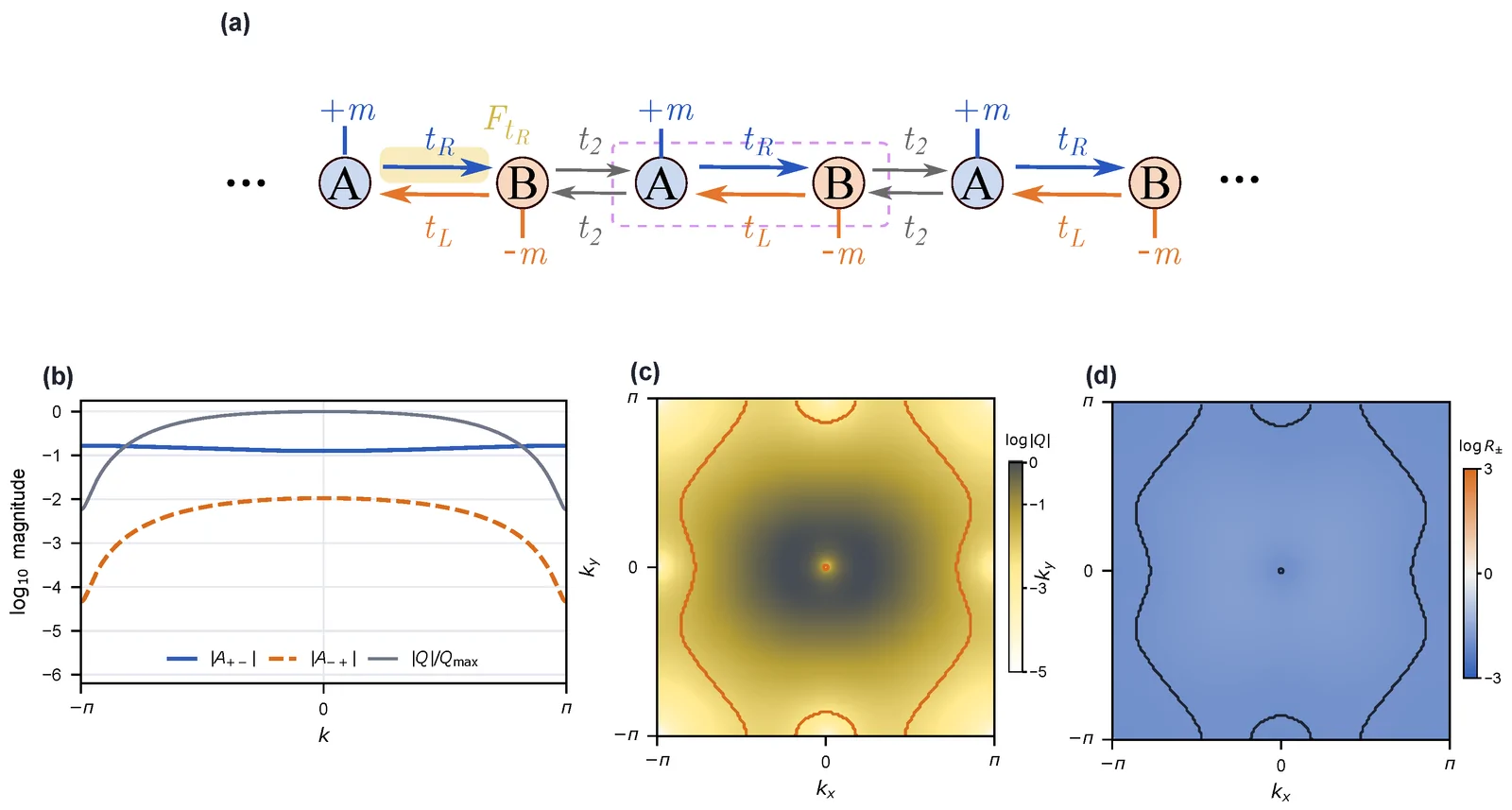
One- and two-dimensional realizations of the local vertex mechanism. (**a**) A nonreciprocal SSH/Rice-Mele chain with a one-sided hopping vertex $F_{t_{R}}$; the purple dashed box marks the highlighted unit cell used to show this local vertex. (**b**) Numerical evaluation for the 1D model, comparing the two ordered interband matrix elements with the scalar readout along the same Bloch path. (**c**) Scalar readout for the two-dimensional complex-spin-orbit Bloch model in the Brillouin zone; the orange curve marks the selected near-null boundary. (**d**) The corresponding ordered-vertex contrast for the same 2D model, evaluated in the fixed biorthogonal convention of Section 2; the black curve marks the same boundary. In both examples the scalar component can be suppressed while one ordered interband matrix element remains finite. The 1D calculation uses Equation (19) with $t_{R}=1.1$, $t_{L}=0.9$, $t_{2}=1$, m=3.0, and 4001 uniformly spaced *k* points. The 2D calculation uses Equations (20)–(23) with M=2.2, λ=0.98, $\left(\eta_{x},\eta_{y}\right)=\left(1,0.7\right)$, $\gamma_{z}=0.25$, and a 201×201$\left(k_{x},k_{y}\right)$ grid. Contours mark $|Q_{vv}^{\left(+\right)}|<10^{-2}\max_{k}\left|Q_{vv}^{\left(+\right)}\right|$; the displayed ordered-vertex contrast is evaluated in the same fixed convention.

Here, the drive is itself a one-sided hopping vertex, making the connection between the lattice perturbation and the local interband vertex explicit. The one-sided modulation $F_{t_{R}}$ places the local interband vertex near a chiral branch of $g_{\pm }$-space. Figure 2a shows this input at the lattice level: the same nonreciprocal SSH chain supplies both the Bloch Hamiltonian and the one-sided hopping perturbation. Figure 2b follows the corresponding Bloch trace. The two ordered interband matrix elements separate while the scalar component is suppressed near the same part of the Brillouin zone. The suppressed scalar component therefore reflects the product contraction of ordered matrix elements generated by a chiral vertex, rather than the disappearance of local interband geometry.

### 4.2. Two-Dimensional Non-Hermitian Complex-Spin–Orbit Lattice

To separate the mechanism from the one-dimensional hopping geometry, we use the two-dimensional lattice-regularized complex-spin–orbit model

(20) $$H_{2D}\left(k\right)=d_{2D}\left(k\right)\cdot \sigma ,$$

which is a non-Hermitian two-band lattice Hamiltonian. Its components are

(21) $$\begin{array}{cc} d_{x} & =\sin k_{x}+i\lambda \sin k_{y}, \end{array}$$

(22) $$\begin{array}{cc} d_{y} & =\sin k_{y}-i\lambda \sin k_{x}, \end{array}$$

(23) $$\begin{array}{cc} d_{z} & =M-\eta_{x}\cos k_{x}-\eta_{y}\cos k_{y}+i\gamma_{z}. \end{array}$$

In the numerical evaluation we use M=2.2, λ=0.98, $\left(\eta_{x},\eta_{y}\right)=\left(1,0.7\right)$, and $\gamma_{z}=0.25$. The anisotropy weakly breaks the x/y symmetry of the lattice regularization. The classification is controlled by the projected vertex in $g_{\pm }$-space, not by this particular anisotropy. The drive is the mass vertex $F_{M}=\sigma_{z}$. It is diagonal in the orbital basis, but its projection in the biorthogonal eigenbasis can lie near a complex-null direction. The relevant quantity is the position of the physical vertex in the local $g_{\pm }$-space, not the microscopic shape of the drive in the orbital basis.

Figure 2c shows the scalar readout produced by this mass vertex across the two-dimensional Brillouin zone. The near-null contours mark where the product entering the scalar component is small. Figure 2d shows the ordered-vertex contrast on the same momentum plane, identifying the parts of the scalar near-null structure that retain a finite ordered interband matrix element in the chosen biorthogonal convention. The two-dimensional example therefore shows that the mechanism is not tied to a one-dimensional hopping vertex.

### 4.3. Three-Dimensional $k_{z}$-Only Stacking Direction

The three-dimensional lattice setting isolates the role of the stacking direction. The non-Hermitian term is confined to the $k_{z}$ stacking dependence, while the transverse $k_{x},k_{y}$ planes retain Hermitian hopping. When the stacking vertex is chiral, this single direction is enough to generate the local ordered-matrix-element contrast. The corresponding Hamiltonian is

(24) $$\begin{array}{cc} d_{x} & =\sin k_{x}+\frac{i\gamma_{z}}{2}\sin k_{z}, \end{array}$$

(25) $$\begin{array}{cc} d_{y} & =\sin k_{y}+\frac{\gamma_{z}}{2}\sin k_{z}, \end{array}$$

(26) $$\begin{array}{cc} d_{z} & =M-\cos k_{x}-\cos k_{y}-t_{z}\cos k_{z}. \end{array}$$

The stacking vertex is

(27) $$F_{\gamma_{z}}=\sin k_{z}\left(\frac{i}{2}\sigma_{x}+\frac{1}{2}\sigma_{y}\right)=i\sin k_{z}\;\sigma_{-},$$

with $\sigma_{-}=\left(\sigma_{x}-i\sigma_{y}\right)/2$. In sublattice language, the non-Hermitian part of the $k_{z}$ stacking is one-sided. Because the transverse planes are Hermitian, the ordered contrast is introduced through the stacking vertex. Replacing this vertex by a diagonal gain/loss-like term removes the single-matrix-element selection in the fixed convention even when scalar near-null regions remain.

**Figure 3:**
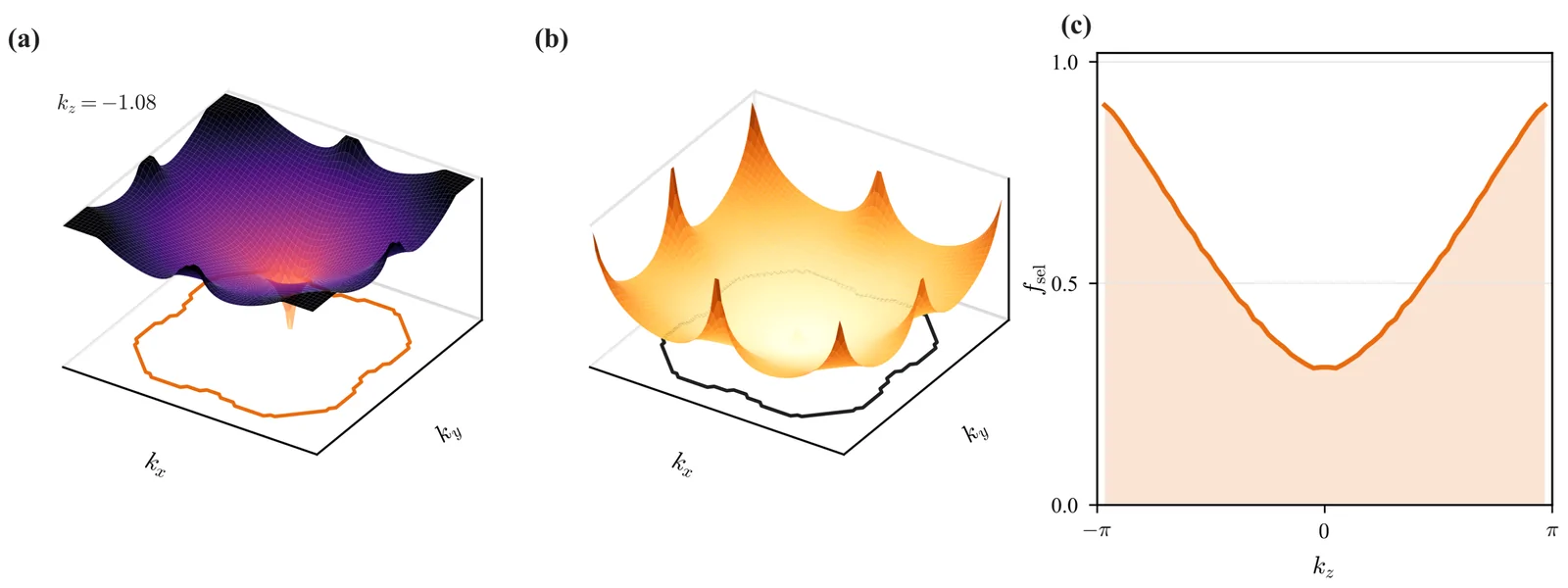
Three-dimensional $k_{z}$-only chiral ladder stack. (**a**) Surface rendering of scalar suppression on a representative $k_{z}$ slice; the color scale gives the normalized scalar magnitude, and the orange curve outlines the selected region on the base plane. (**b**) Surface rendering of the ordered-vertex contrast on the same slice and in the same biorthogonal convention; the color scale gives the fixed-convention contrast, and the black curve marks the selected-region boundary. (**c**) Fraction $f_{\mathrm{sel}}$ of selected $\left(k_{x},k_{y}\right)$ points as a function of $k_{z}$; the orange curve gives this fraction. The three views show that the scalar near-null region can coincide with a finite ordered interband matrix element over an extended momentum-space support. The calculation uses Equations (26) and (27) with M=2.4, $t_{z}=1$, $\gamma_{z}=0.75$, on a $61^{3}$ uniform Brillouin-zone grid; panels (**a**,**b**) use the slice $k_{z}=-1.08$. The outlined region satisfies $|Q_{vv}^{\left(+\right)}|<10^{-2}\max_{k}\left|Q_{vv}^{\left(+\right)}\right|$ and an ordered-matrix-element ratio larger than 20 in the fixed biorthogonal convention.

Figure 3 displays this three-dimensional result in the same local convention. Figure 3a shows a representative $\left(k_{x},k_{y}\right)$ slice of the scalar component at fixed $k_{z}$; the outlined regions are scalar near-null loci of the local product. Figure 3b keeps the same slice and displays the ordered imbalance, showing whether the near-null loci also carry an asymmetric interband matrix element in the same biorthogonal convention. Figure 3c shows the fraction of selected points in each $k_{z}$ slice, demonstrating that the effect is not confined to an accidental two-dimensional cut. Thus an extended scalar near-null region can still support a nonzero ordered interband matrix element.

The matched diagonal $\gamma_{z}$-stack keeps the same Hermitian transverse planes and the same $k_{z}$-dependent non-Hermitian direction but replaces the chiral $\gamma_{z}$ dependence by a diagonal one:

(28) $$\begin{array}{cc} H_{\mathrm{diag}}\left(k\right)= & \sin k_{x}\;\sigma_{x}+\sin k_{y}\;\sigma_{y}\\ \; & +\left[M-\cos k_{x}-\cos k_{y}-t_{z}\cos k_{z}\right.\\ \; & \;\left.+\;i\gamma_{z}\sin k_{z}\right]\sigma_{z}. \end{array}$$

Taking the derivative of Equation (28) gives the physical vertex

(29) $$F_{\gamma_{z}}^{\mathrm{diag}}=\partial_{\gamma_{z}}H_{\mathrm{diag}}=i\sin k_{z}\;\sigma_{z}.$$

A similar scalar near-null structure can appear without single-matrix-element selection. The comparison isolates the relevant ingredient: the stacking vertex must be chiral after projection to the local interband $g_{\pm }$-space. For a chiral $k_{z}$ vertex, the scalar near-null region coincides with one suppressed ordered matrix element, whereas the diagonal $k_{z}$ vertex leaves the ordered matrix elements unselected in the same convention.

## 5. Discussion and Conclusions

The classification separates chiral-vertex selection from other sources of a small scalar component. In a Hermitian two-band problem, the two off-diagonal matrix elements are conjugate-locked; invariantly, one of them vanishes if and only if the other one vanishes. A generic non-Hermitian perturbation can change the relative magnitudes of the two ordered matrix elements, but a scalar near-null component by itself does not imply selection of an ordered matrix element. The chiral case appears when the interband part of the physical vertex approaches a complex-null branch, $g_{+}≃0$ or $g_{-}≃0$, in the local biorthogonal eigenbasis. This step goes beyond the product identity itself. The physical vertex decides which point of the local $g_{\pm }$-space is sampled.

The lattice models test this distinction in one-, two-, and three-dimensional Bloch settings. The nonreciprocal SSH/Rice-Mele chain gives the minimal one-dimensional realization through a one-sided hopping vertex. The two-dimensional complex-spin–orbit lattice shows that the same $g_{\pm }$-space mechanism is not tied to a one-dimensional hopping perturbation. The $k_{z}$-only ladder stack gives a controlled three-dimensional comparison: a chiral stacking vertex produces an ordered contrast near a scalar near-null region in the chosen biorthogonal convention, whereas a diagonal $k_{z}$ gain/loss stack can keep the ordered matrix elements unselected even though the scalar component is small.

The result is a local statement about the geometric response kernel of a spectrally separated, diagonalizable two-band block away from exceptional points. A device conductance, admittance, or non-Bloch boundary response requires a specification of occupations or reservoirs, boundary conditions, drive normalization, loss normalization, and a measurement protocol [66,67,68,69,70,71,72]. These ingredients determine how the local vertex kernel is embedded in a transport or boundary problem.

The ordered matrix elements also give a controlled way to discuss possible measurements without specifying a full device. A modulation of hopping, onsite detuning, strain, gain and loss, or Peierls phase selects the physical vertex, while a source/readout protocol determines which left–right ordering is probed. In this limited sense, the local geometric kernel has a product-contraction structure: the scalar product can vanish while the resolved pair $\left(g_{+}^{\left(v\right)},g_{-}^{\left(v\right)}\right)$ identifies which ordered matrix element is suppressed. This parallels ordering effects known from non-Hermitian dynamics, including asymmetric mode switching and asymmetric nonadiabatic transitions around exceptional points [73,74], but here the ordering is resolved inside the local QGT vertex contraction. The distinction between nonorthogonal eigenstate effects and exceptional-point proximity also appears in recent work on non-Hermitian state discrimination and information flow [75]. This provides a local organizing principle for platforms in which drive vertices and readout channels can be controlled separately, such as photonic, circuit, mechanical, and active-matter realizations [20,21,62,63,64,65].

Several extensions require additional structure. In a multiband problem, the scalar component contains a sum over intermediate bands. The two-factor classification then applies directly only after a spectrally separated two-band block or a dominant intermediate contribution has been isolated. Near an exceptional point, small denominators can enhance both ordered matrix elements; this gap-driven amplification is distinct from a chiral zero of one $g_{\pm }$ coordinate. In a nonreciprocal lattice, the present Bloch analysis evaluates the local vertex in momentum space, whereas a full open-boundary or non-Bloch response also depends on the boundary and measurement structure described above.

In summary, the scalar biorthogonal QGT component along a specified physical drive of a spectrally separated non-Hermitian two-band model factorizes into two ordered interband matrix elements. The pair $\left(g_{+}^{\left(v\right)},g_{-}^{\left(v\right)}\right)$ contains the local interband content of the physical perturbation, while the scalar component retains only the product. A null or near-null scalar component can therefore have different origins: no interband geometry, Hermitian locking, gain/loss-type responses without ordered-matrix-element selection, or a complex-null vertex with one suppressed ordered matrix element and a finite reverse element. The last case is the local chiral-vertex mechanism identified here.   

## Figures and Tables

**Table 1:** Local vertex classes for spectrally separated, diagonalizable two-band models. The Hermitian row is a symmetry-restricted slice: the gauge-invariant statement demonstrates zero-locking of the opposite ordered matrix elements, while the displayed conjugate form assumes a Hermitian unitary convention.

Class	Vertex-Space Meaning
0	$g_{+}=g_{-}=0$: no interband geometry, realized by identity or longitudinal vertices.
1	Hermitian-locked slice: in a unitary convention $g_{-}={\left(g_{+}\right)}^{*}$; invariantly, $g_{+}=0\Leftrightarrow g_{-}=0$.
2	$g_{+}g_{-}\neq 0$: generic complex-transverse vertex with finite scalar readout.
3	$g_{+}=0,\;g_{-}\neq 0$: chiral branch with scalar-null readout and one nonzero ordered matrix element.
4	$g_{-}=0,\;g_{+}\neq 0$: opposite chiral branch with the ordered labels interchanged.

## Data Availability

The data supporting the findings of this study are contained within the article. Numerical scripts and additional arrays used for figure generation are available from the corresponding author upon reasonable request.
